# Starch Properties of Roasting Rice from Naturally High-Resistant Starch Rice Varieties

**DOI:** 10.3390/molecules28176408

**Published:** 2023-09-02

**Authors:** Ruifang Yang, Jianhao Tang, Qi Zhao, Zhongze Piao, Gangseob Lee, Changzhao Wan, Jianjiang Bai

**Affiliations:** 1 Key Laboratory of Germplasm Innovation and Genetic Improvement of Grain and Oil Crops (Co-Construction by Ministry and Province), Ministry of Agriculture and Rural Affairs, Crop Breeding and Cultivation Research Institute, Shanghai Academy of Agricultural Sciences, Shanghai 201403, China; yangruifang1982@163.com (R.Y.); tangjianhao@saas.sh.cn (J.T.); 20230203@saas.sh.cn (Q.Z.); piaozhongze@126.com (Z.P.);; 2 Department of Agricultural Biotechnology, National Institute of Agricultural Sciences, Rural Development Administration, Jeonju 54874, Republic of Korea; kangslee@korea.kr

**Keywords:** roasting, resistant starch, rice, digestion rate

## Abstract

This study investigates the effects of moisture content control on the characteristics, properties, and in vitro starch digestion of roasted rice powder made from natural high-resistant starch (RS) rice varieties. The results demonstrate that adjusting the moisture content before roasting significantly affects the RS content of the roasted rice powder. Among various moisture levels tested, the addition of 15% water (rice-to-water ratio of 85:15) before roasting resulted in the highest RS content, reaching 22.61%. Several key parameters of the rice samples before and after optimal moisture control were analyzed, including thermal stability, chain length distribution, volatile flavor composition, and scanning electron microscopy. Additionally, in vitro digestion properties were measured. The findings revealed that the volatile flavor compounds in the high-RS roasted rice significantly increased compared to non-roasted rice. Moreover, the thermal stability of the rice samples improved, and the chain length distribution exhibited significant changes. The water absorption and expansion properties were significantly lower in the high-RS roasted rice. Furthermore, the in vitro starch digestion of the roasted flour made from high-RS rice showed a significantly lower digestion rate compared to common rice, indicating a lower starch hydrolysis index in high-RS rice with the *sbe-rs* genotype. Overall, the roasting process of natural high-RS rice modifies its characteristics, increases the RS content, enhances the flavor, and results in a lower starch digestion rate compared to common rice. This study provides valuable data for the food industry to promote the application of high-RS rice varieties with mutations in the *SBEIIb* gene, such as Youtangdao2 (YTD2).

## 1. Introduction

*Diabetes mellitus*, a group of metabolic diseases characterized by high levels of blood glucose, is one of the major diseases that pose significant health risks [1]. According to the data released by the International Diabetes Federation in 2021, about 537 million adults (20–79 years old) in the world are suffering from diabetes, and China is the country with the largest number of diabetics. Diabetes-related complications and comorbidities result in a significant global economic burden [2]. In China alone, the diabetic population has already reached 141 million in 2021, and more than 500 million individuals are in the pre-diabetic stage. A scientific and reasonable diet is the key to preventing this disease [3]. Resistant starch (RS), a healthy dietary fiber, also known as indigestible starch, cannot be enzymatically digested in the small intestine, but can be fermented into a series of short-chain fatty acids (SCFAs) in the large intestine [4,5,6]. RS has very important physiological functions, such as controlling postprandial blood sugar, preventing and treating intestinal diseases, lowering lipids, controlling body weight and promoting mineral absorption, and so on [7,8].

In recent years, RS has become a hot topic in functional rice research and many high-RS rice mutants or varieties have been identified [9,10,11,12,13,14,15,16]. Rice is generally considered to be a high Glycemic index (GI) food [17], the content of RS in high-quality rice is only 0.1–0.5%, and is generally under 3% in hot-cooked rice varieties [18]. Jiangtangdao 1 is a japonica variety rich in RS independently bred by Shanghai Academy of Agricultural Sciences (SAAS). In a previous study, we located a putative gene, *sbe3-rs*, in mutant Jiangtangdao1, which contained a missense mutation, Leu-599-Pro, in *OsSBEIIb* [19]. The content of RS in raw milled rice of Jiangtangdao1 is up to 10%, dozens of times that of ordinary rice, and its commodity name is Youtang rice. Our previous research showed that the GI of Youtang rice is 48.53, which is a kind of low GI food [20]. Many studies have shown that postprandial blood glucose levels in type 2 diabetes patients who eat high RS rice fluctuate less than those who eat ordinary rice in the real world and take hypoglycemic drugs and simulate ordinary diet, and their blood glucose changes are significantly reduced 30 min and 60 min after eating high RS rice. In addition, the incremental area under the blood glucose curve of diabetics who ate high-RS rice was smaller than that of regular rice [21]. Our research results are consistent with those of previous studies. The postprandial blood glucose of type II diabetic patients fed with Youtang rice was significantly lower than that of ordinary rice, and long-term consumption of Youtang rice significantly improved glycosylated blood red egg white (HhAlc), triglyceride (TG), high density fat egg white (HDLC), low density lipoprotein (LDLC) and serum resistin concentrations in type II diabetic patients, but there was no statistical significance [22,23].

There are five different types of RS (RS I-V). RS I refers to starch that is physically embedded, such as within a protein matrix or thick cell wall. RS II represents raw granules or ungelatinized starch. RS III refers to retrograded starch, which is formed through the process of cooling and solidification after cooking and then cooling again. RS IV indicates chemically or enzymatically modified starch. Finally, RS V represents lipid–starch complexes, where starch is associated with lipids [24]. These classifications categorize the different forms of RS based on their physical and chemical properties. Previous studies have found that the contents of RS are different in different processing methods [10]. Although Youtang rice has shown great function in controlling blood sugar after meals, its popularization and utilization are limited by its high amylose content, hard texture and poor eating quality [25]. The commonly used processing methods of grain raw materials include conventional roasting, infrared baking, boiling and extrusion. It has been previously reported that the RS content of the freshly cooked *sbeIIb* rice was about 5–9% [26]. Roasting is a typical food processing operation involving dry heating, and is a common tool to improve the flavor and shelf-life of materials [27,28,29]. In our previous study, we investigated the effect of moisture content on the RS content of Youtang rice. Before the roasting process, we obtained a wide range of moisture content levels by soaking the rice and subjecting it to continuous drying. The moisture content levels ranged from 13% to 38% of rice. The findings indicated that Youtang rice exhibited a significant increase in RS content of over 20% following a specific roasting procedure when the moisture content was around 20% before roasting [25]. Based on the international standard ISO 26642:2010 for determining the glycemic index (GI) of food products and providing recommendations for food classification, we collaborated with the China National Research Institute of Food and Fermentation Industries in Beijing, China. The calculated GI value for roasted Youtang rice flour was found to be 56. These findings were obtained through a rigorous adherence to standardized protocols for food testing and bioanalytical measurements. The high-RS content rice powder obtained by roasting Youtang rice can be used as meal replacement powder, energy bar and other main base material suitable for people to control blood sugar.

This study aims to investigate the impact of adjusting the rice-to-water ratio before roasting, which affects moisture content and is relatively easy to implement, on the characteristics and in vitro starch digestion of roasted rice powder derived from natural high-RS rice varieties. The primary focus of this research is to analyze the changes occurring during the roasting process, including the increase in RS content and the enhancement of flavor. Additionally, this study aims to provide valuable insights into the practical application of high-RS rice varieties resulting from mutations in the OsSBEIIb gene, such as *sbe3-rs*.

## 2. Results and Discussion

### 2.1. Morphological Characteristics of Starch Grains in High-RS Rice

**Figure 1:**
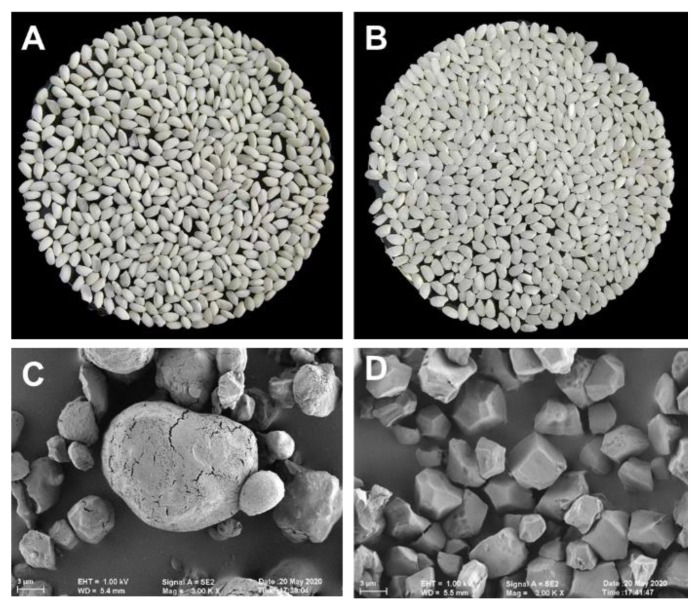
The appearance and scanning electron microscope of high-RS rice YTD2 (**A**,**C**) and common rice HDXR34 (**B**,**D**).

High-RS rice exhibits distinct visual characteristics, including a higher proportion of chalky white grains and an overall increased chalkiness, as presented in Figure 1A. Scanning electron microscopy (SEM) observations further highlight the distinguishing features of the starch granules in high-RS rice. These granules appear to possess indistinct edges and corners, implying a less defined structure (Figure 1C). In contrast, the starch granules in the control rice HDXR34 display a polygonal shape with uniform sizes. The surface of these granules appears smooth and refined, lacking any noticeable cracks or irregularities (Figure 1D).

### 2.2. Resistant Starch Content in High-RS Rice Youtangdao after Roasting

The results of our previous study showed that different processing methods had a great impact on the contents of RS, such as high-pressure bulking, extrusion bulking, making into rice cakes and rice noodles. Compared with unprocessed rice flour (13.6%), the contents of RS were greatly reduced to about 3% [30]. The RS content of gelatinized heat-moisture treatment of *sbeIIb*/*Lgc1* rice native flours was only 3.3% [31]. These results showed that the RS content of high-RS rice with *SBEIIb* mutation gene is easily destroyed by heat and has poor stability, so we need to find a suitable processing method to maintain the RS content to the maximum and improve the taste.

Based on the results presented in Table 1, we can conclude that the moisture content plays a significant role in the formation of RS. For the YTD2 sample, the RS content gradually increases with increasing moisture level. The highest RS content is observed at 15% moisture level, reaching 22.61% (±1.21) (Table 1). However, when the moisture level further increases to 30%, the RS content significantly drops to 8.42% (±0.15) (Table 1). This suggests that there is an optimal moisture level in the roasting process that maximizes the formation of RS in the YTD2 variety. High moisture levels have a negative effect on RS formation. In contrast, for the HDXR34 sample, the RS content remains very low regardless of the moisture level. The RS content of HDXR34 ranges from 0.041% to 0.075% (Table 1), which is much lower than that of YTD2. This indicates that the moisture level before roasting of common rice HDXR34 does not have a significant impact on the RS content during the roasting process. Further research is needed to explore the mechanism by which different moisture levels affect the formation of RS in high RS rice varieties during the roasting process. These findings have important practical implications for the development of rice products with high RS content. In contrast, the effect on the RS content of ordinary rice samples was less pronounced (Table 1). It has been previously reported that the RS content of the cooked *sbeIIb* mutant rice was about 5–9%. The RS content through roasting processing is more than 20%, which is much higher than that of cooking.

Roasting can significantly reduce the moisture content of both high-RS rice samples and common rice samples (Table 1). The moisture content of raw rice is generally below 14% according to the Chinese National Standard GB/T 13587-2017 “Grading of Rice and Rice Quality”. After roasting, it was observed that the moisture content decreased by approximately 3~4% (Table 1). This reduction in moisture content is attributed to the evaporation of water from the sample during the roasting and toasting process [32].

Overall, the findings suggest that roasting treatment may be an effective way to increase the RS content significantly by controlling the water content of rice before roasting. During the roasting process, the main changes are the increase in temperature and the evaporation of moisture. These changes may lead to the denaturation and recrystallization of starch, resulting in the formation of RS. However, the excessive moisture content may lead to excessive steam generation during roasting, potentially causing partial starch gelatinization and inhibiting the crystallization and formation of RS. Conversely, insufficient moisture content may prevent sufficient heat transfer during roasting, thereby failing to reach the temperature necessary for starch crystallization and the formation of resistant starch. The specific mechanism needs to be further analyzed. We further compared and analyzed the physicochemical properties of roasted rice samples with the highest RS content (22.61%) (YTD2-85%) and before roasting, providing guidance for subsequent development.

### 2.3. Changes in Flavor before and after Roasting

Previous studies have demonstrated that roasting can result in the production of Maillard reaction products, such as furans and pyrazines [33]. Our study corroborates these findings, as we observed that the relative content of pyrazine substances increased significantly after roasting, accounting for 29.43% of the total relative content of substances. Before roasting, however, the quantity and relative content of pyrazine substances were negligible at 0% (Table 2). These results suggest that the roasting process of rice is responsible for the generation of pyrazine substances, which contribute to the aromatic profile of roasted rice. These findings align with previous research in this field. Furthermore, before roasting, the category table of substances contained two acid substances, accounting for 43.55% of the relative content. After roasting, the number of acid substances increased to three, while the relative content decreased to 2.68%. This suggests that roasting rice leads to the decomposition and reduction in some heat-sensitive acid substances. These results indicate that the roasting process of rice can cause chemical reactions and produce new compounds. These compounds contribute to the aroma and taste of roasted rice. The possible reason is that the chemical composition of rice changes during the roasting process, resulting in the formation of new compounds such as pyrazine substances. These products can be produced through oxidation, reduction, dehydration, decomposition, and other reaction pathways. After being roasted, the category table of substances exhibits a significant increase in their reaction pathways. Previous studies have indicated that heavy roasting degrees result in the formation of furfural, isoctyl alcohol, and other flavor substances that can cause irritation. On the other hand, medium roasting has been identified as the optimal degree for achieving the best quality characteristics in rice roasting [34]. Therefore, it is very important that the samples in our study are medium roasted until the rice grains are golden, so as to avoid Browning.

### 2.4. Thermal Property

The gelatinization parameters of various rice flours are listed in Table 3. Based on the DCS thermal stability test results, it is evident that roasting the high-RS rice significantly increased the ΔH value from 2.893 J/g to 3.653 J/g. This indicates an increase in the heat absorbed or released per gram of the sample during thermal decomposition after roasting. Furthermore, the Peak (Tp) gelatinization temperature rose from 77.2 °C to 85.4 °C, indicating an elevated maximum peak temperature of particle thermal decomposition following roasting. Additionally, the onset (To) and conclusion (Tc) values increased from 67.0 °C to 76.6 °C and from 86.1 °C to 92.8 °C, respectively, indicating enhancements in the sample’s initial temperature of thermal decomposition and high-temperature thermal stability after roasting. The Tc temperature of ordinary rice is usually around 70 degrees [35]. However, the Tc of high-RS rice will usually exceed 80 degrees. Because high-RS rice is different from ordinary rice in structure, its gelatinization characteristics will also change, so there will be a higher Tc in DCS tests. This temperature difference can also be used to distinguish between different types of rice.

In conclusion, the results from the DCS thermal stability test demonstrate that the roasting process positively affects the thermal stability of rice particles. This can be attributed to the gelatinization of starch molecules during the baking process. The gelatinized starch molecules form a more stable structure, enabling them to withstand high temperatures more effectively and thereby enhancing the thermal stability of rice. The enhanced thermal stability of roasted rice signifies its improved resistance against physical and chemical transformations during high-temperature processing and storage, leading to increased efficiency in food processing, extended shelf life, and improved sensory attributes.

### 2.5. Water Solubility Index and Swelling Power

The starch solubility index and swelling power of high-RS rice before and after roasting were determined and the results are presented in Table 4. The starch solubility index of high-RS before roasting was found to be higher (37.42%) than that of after roasting (24.63%). This suggests that the before-roasting sample has a higher ability to dissolve in water compared to the after-roasting sample. The swelling power of high-RS before roasting (13.55 g/g) was also found to be higher than that of after roasting (8.39 g/g). This indicates that the high-RS rice before roasting has a higher ability to expand when subjected to thermal treatment, which is likely due to the presence of intact starch granules in the unroasted sample.

In summary, the results of this study suggest that the water solubility and expansion properties of high-RS rice samples are affected by the roasting process. Before roasting, rice has a higher starch solubility index and swelling power compared to after roasting, which could have implications for their use in various food applications.

### 2.6. Amylopectin Chain Length Distribution

**Figure 2:**
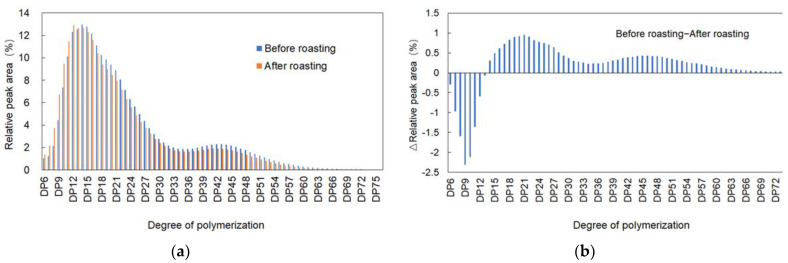
Comparison of chain length distribution of amylopectin of Youtang Rice with high-RS content before (**a**) and after roasting (**b**).

It can be seen that the degree of polymerization (DP) of the samples after roasting significantly increases within the DP6-DP12 range, reaching a peak around DP9. Furthermore, the polymerization degree of the longer chains, particularly DP13 and above, is significantly lower in the samples after roasting compared to before roasting, with the difference reaching its peak at DP21 (Figure 2). Previous study shows that the amylopectin of high-RS mutants have more long-chain glucans, making the texture of rice grains very hard and nonsticky after boiling [19,36]. These results indicate that the roasting process has an impact on the molecular structure of starch and may improve the palatability of high-RS rice.

Generally, during heating, starch molecules can undergo partial hydrolysis and degradation, leading to a decrease in chain length. Due to the high-temperature heating involved in roasting, this can result in the fragmentation or shortening of certain starch chains, particularly the longer chains. The roasting process significantly affects the molecular structure of starch, resulting in an increase in short-chain starch and a decrease in long-chain starch. These structural changes may have an impact on the digestibility and texture of the roasting samples.

### 2.7. Molecular Weight Distribution of Debranched Starch

The molecular weight distribution of debranched starch was determined by gel permeation chromatography (GPC). Peak1 consisted of short chains of amylopectin, Peak2 consisted of medium chains of amylopectin and, Peak3 consisted of amylose, respectively [37]. GPC results listed in Table 5 showed that the area of Peak1 relative to the total area increased from 47.99% to 52.51% after roasting. The area of Peak2 relative to the total area decreased from 35.56% to 31.49%. Peak3, on the other hand, remained relatively stable, with an area of approximately 16.45% ± 0.1% before roasting and 16.0% ± 0.2% after roasting. These results suggest that the molecular weight distribution of debranched starch underwent some changes during the roasting process. The increase in the area of Peak1 indicates the formation of more high molecular weight components after roasting. The decrease in the area of Peak2 suggests some degradation or transformation of medium molecular weight components during roasting. The relatively stable area of Peak3 implies a lesser impact on the low molecular weight components. These changes in the results could be attributed to the chemical reactions and degradation of starch during the roasting process. Factors such as temperature and duration of roasting may induce the degradation and re-aggregation of starch molecules, thereby affecting the molecular weight distribution. Additionally, roasting may also involve other chemical reactions such as gelatinization and enzymatic activities, further influencing the starch’s molecular weight distribution.

**Figure 3:**
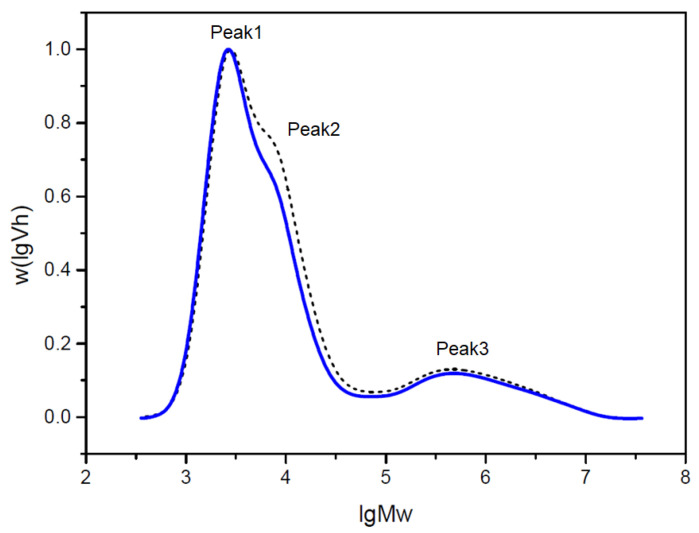
Molecular weight distribution of high-RS rice before and after roasting. Dotted line indicates before roasting, solid line indicates after roasting. The areas of three peaks Peak1, Peak2 and Peak3 represent the contents of medium and short chain of amylopectin (Ap1), long chain of amylopectin (Ap2) and amylose (Am), respectively.

We used X-ray diffraction analysis in the early stage and found that the relative crystallinity of starch in high-RS rice after roasting was 46.68%, which was slightly higher than that of 41.36% before roasting [25]. This may lead to a further increase in the content of resistant starch. At the same time, Peak1 increased and Peak2 decreased through GPC analysis (Figure 3). There is some correlation between the two findings. These findings provide insights into the structural and property changes of starch during the roasting process, and further research can delve deeper into understanding the mechanisms behind the influence of roasting on starch of high-RS rice.

### 2.8. In Vitro Starch Digestion of Sample before and after Roasting

**Figure 4:**
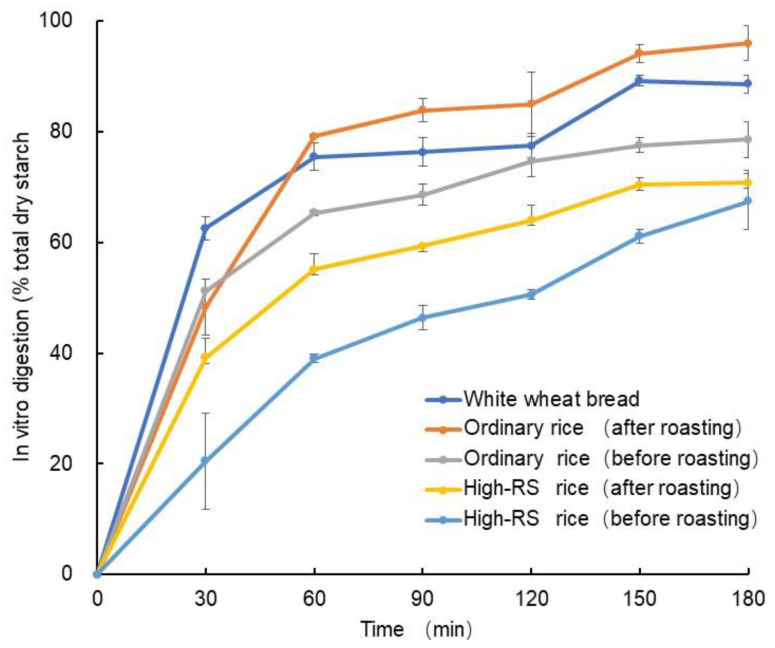
In vitro digestion of starch in high-RS content rice and ordinary rice before and after roasting. Control: white wheat bread.

Rice has high GI because of its high starch content and is associated with the development of diabetes [38]. The starch hydrolysis index was measured at various time points for all the samples. It was observed that the starch hydrolysis index of white wheat bread and ordinary rice samples increased over time (Figure 4). This finding is consistent with previous studies [39,40], suggesting that these samples are more readily digested. In contrast, the starch hydrolysis index for high-RS samples was significantly lower than the control and ordinary rice samples, indicating that high-RS is harder to digest. On the other hand, the starch hydrolysis index of the samples before roasting was lower than that of the samples after roasting. Roasting increased the starch hydrolysis index of both ordinary rice and high-RS rice, which suggests that this process can increase the digestibility of these grains slightly. These results align with the properties of high-RS, which has been shown to be more resistant to digestion [8,9,40]. Therefore, our findings suggest that selecting foods based on their type of starch and processing methods is essential for optimal nutrition and health.

To consume high-RS roasted flour, it is typically mixed with boiling water to create a thick paste-like consistency. This step is essential to ensure proper texture and enhance the eating experience. By adding boiling water, the rice flour absorbs moisture, forming a thick and smooth consistency that is easier to consume and digest. Our study revealed that the RS content of roasted rice flour was as high as 20% when it was not mixed with boiling water, but decreased to about 10% after boiling water was added [25]. This reduction in RS content can be attributed to the poor thermal stability of resistant starch, which leads to its degradation during the boiling process. To maximize the beneficial effects of high RS roasted rice flour and improve its stability, further investigation is needed to develop meal replacement formulations using this rice flour as the main ingredient. Additionally, exploring the use of lower temperatures may help mitigate the loss of RS and enhance the thermal stability of the roasted rice flour in such meal replacement products.

## 3. Materials and Methods

### 3.1. Materials

The high-RS rice variety Youtangdao 2 (YTD2) bred by the Shanghai Academy of Agricultural Sciences, Shanghai, China, has a RS content of up to ~13%. The RS content was nearly 20 times higher than in common rice, which was mainly caused by a single amino acid mutation of *OsSBEIIb* that reduced the activity of the rice starch branching enzyme [19]. Ordinary elite japonica rice cultivar Hudaoxiangruan34 (HDXR34) with normal RS (~0.5%) was used as a common rice control. YTD 2 and HDXR34 were planted in the field during the growing season in 2022 in Shanghai, China.

### 3.2. Scanning Electron Microscope

The rice was first thoroughly rinsed and then soaked in double-distilled water overnight to ensure proper hydration (the rice-to-water ratio was 1:3). Subsequently, the softened rice was meticulously ground into a smooth paste, which was then immersed in a 0.4% sodium hydroxide solution for precisely 24 h. Following this treatment, the rice paste underwent neutralization with hydrochloric acid and was thoroughly washed with water in a sequence of five repetitions. This series of steps contributes to the removal of unwanted impurities. To concentrate the desired components, the resulting mixture was subjected to centrifugation at 4000 rpm, yielding a valuable precipitate. This precipitate was carefully dried at a controlled temperature of 40 °C for a duration of two days in an oven (DHG-9055A; Yiheng, Shanghai, China), ensuring complete removal of moisture. After drying, the resulting material was finely ground into a powdered form using a lab-scale grinder (Jiupin Industry and Trade Co., Ltd., Jinhua, China) and sieved using a 100-mesh size to obtain a uniform particle size of 150 µm. The powdered starch was then appropriately stored at a temperature of 4 °C to maintain its integrity and composition. To facilitate observation and analysis, a small quantity of the starch sample was dispersed in anhydrous ethanol and carefully placed onto a cover glass. This prepared sample was then securely attached to an aluminum alloy specimen holder, followed by a thin coating of gold using a sputter-coating technique. The observation was performed at a magnification of 3000×, utilizing an accelerating voltage of 20 kV and maintaining a working distance of 10 mm.

### 3.3. Roasting Rice Flour Preparation

Distilled water was added to the milled rice in various proportions to create different fractions of rice-to-water ratios: 100% (200 g milled rice), 90% (200 g milled rice/22 g water), 85% (200 g milled rice/35 g water), 80% (200 g milled rice/50 g water), and 70% (200 g milled rice/85 g water). After the addition of water, the rice was left for a minimum of one hour to ensure complete absorption at room temperature (RT) and relative humidity at 40–60%. This step allowed the rice to fully hydrate and soften before proceeding with further processing. Subsequently, the hydrated rice was roasted until it reached a golden-brown color. The roasting process was carried out using a small electromagnetic stir-frying machine, maintained at a temperature of 150 °C for a specific duration ranging from 30 to 50 min. Once adequately roasted, the rice was crushed using an electric grinder and passed through a 100-mesh sieve to obtain a fine and uniform texture.

### 3.4. Moisture Content

To determine the precise moisture content of the roasted rice, an Ohaus MB90 Moisture Analyzer (Parsippany, NJ, USA) was employed by desiccation of a 2 g sample for 15 min at 105 °C, based on the principle of the oven drying method for moisture determination. This equipment provided accurate measurements of the residual moisture present in the rice after the roasting process.

### 3.5. Resistant Starch Content

The method used to determine the RS content in rice was the AOAC method 2002 02, using the RS analysis kit from Megazyme (K-RSTAR 08/11, Wicklow, Ireland). Briefly, 0.1 g of rice flour was incubated with amylase and starch glucosidase for 16 h at 37 °C. This enzymatic digestion broke down the digestible starch in the rice into glucose, leaving behind the RS. After digestion, an equal volume of ethanol was added to the digest to precipitate the RS. The mixture was centrifuged, and the pellet was washed twice with 50% ethanol. The RS was then dissolved in 2 M KOH and neutralized with acetate buffer. Starch glycosidase was added to hydrolyze the RS to glucose. Finally, the glucose oxidase/peroxidase reagent was added to the sample for color development, and the absorbance of each solution was measured at 510 nm using a spectrophotometer. The glucose concentration was then converted to RS content.

### 3.6. Apparent Amylose Content

Apparent amylose content (AAC) was determined using the iodine spectrophotometric method according to the standard of Chinese Ministry of Agriculture, NY/T 2639-2014. Briefly, 100 mg flour was suspended in ethanol (1 mL) and sodium hydroxide (9 mL, 1 N). The suspension was then heated (100 °C, 10 min) to gelatinize the starch. The samples were then cooled to room temperature and 5 mL of the volume of the suspension was made up to 100 mL with deionized water. Then, 1 mL of acetic acid (1 M) was added to the volumetric flask to acidify the sample, 1.5 mL of iodine solution was added, the mixture was shaken well and the sample was left for 20 min to allow the reaction to occur. The absorbance of each sample was measured at 620 nm using a spectrophotometer. The analysis was triplicated for each sample treatment replicates.

### 3.7. Determination of Fragrance Substances

Gas chromatography-mass spectrometry (GC-MS) was used to extract, identify and analyze the flavor compounds of unroasted and roasted rice samples. The method used refers to Yao et al. [41]. The procedure involved crushing and placing 5 g of samples into a 20-mL headspace vial. Following the addition of sodium chloride, the vial was sealed and heated in a water bath at 80 °C for 30 min. Afterward, a solid-phase microextraction needle (57348-U, Supelco, Bellefonte, PA, USA), pre-aged at 250 °C, was inserted into the vial for a 30-min extraction period. The needle was then immediately inserted into the GC inlet for desorption, which lasted for 3 min. Subsequently, volatile compounds were analyzed using a GC-MS system (Agilent 7693-7000D, Santa Clara, CA, USA) equipped with an HP-5MS chromatographic column (60 m × 250 μm × 0.25 μm). The heating procedure was as follows: the initial temperature was maintained for 3 min, and then increased to 250 °C at the rate of 6 °C/min for 5 min. The collected mass spectra from the analysis were compared with the NIST spectrum library to facilitate the identification of volatile components present in the samples. The relative contents of each identified component were then determined using the area normalization method, allowing for a quantitative analysis of the various fragrance substances.

### 3.8. Differential Scanning Calorimetry (DCS)

The gelatinization characteristics of starch granules were determined using differential scanning calorimetry (DSC, TA Instruments Q2000, New Castle, DE, USA). The sample was prepared according to the procedure of Wang et al. with a slight modification [42]. In brief, a precise weight of 5 mg of rice flour, previously defatted with petroleum ether, was measured and placed into an aluminum pan. Subsequently, 15 μL of deionized water was added to the pan. The pan was hermetically sealed to prevent any moisture loss and allowed to equilibrate at room temperature for a duration of 1 h. Following the equilibration period, the sample was subjected to heating at a constant rate of 10 °C/min, starting from 30 °C and progressing up to 105 °C. Use Universal Analysis/Proteus Thermal Analysis software for data analysis. The onset temperature (To), peak temperature (Tp), crystallization temperature (Tc) and enthalpy of gelatinization (ΔH) were calculated to characterize the phase transition process of the sample, repeated three times per sample. These parameters provide insights into the timing and intensity of gelatinization, allowing for a better understanding of the thermal properties of the starch granules.

### 3.9. Water Solubility Index and Swelling Power

According to the method of Wang et al. [42] the water solubility index and swelling force of the third solution were determined with slight modification. An appropriate amount of sample (about 0.1 g) was weighed into a test tube, and the mass of the empty test tube was recorded as M1. Then, 10 mL of deionized water was added to the test tube. The test tube was shaken on an oscillator for 10 s to ensure the starch solution was in a suspended state. Subsequently, it was placed in a 95 °C water bath for 60 min, with gentle shaking every 10 min. Afterward, the test tube was cooled to room temperature in an ice bath and centrifuged at 8000× *g* for 20 min. A clean aluminum tray was taken and dried at 80 °C for 1 h. It was then removed from the oven and placed in a constant temperature and humidity box for 20 min. The tray was weighed and recorded as M3. The supernatant was poured into the aluminum tray, while the test tube containing the precipitate was dried at 80 °C for 20 min. After drying, it was taken out and placed in a constant temperature and humidity box for 20 min. The test tube was weighed and recorded as M2. The supernatant in the aluminum tray was dried to a constant weight in an 80 °C oven. It was then removed from the oven and placed in a constant temperature and humidity box for 20 min. The dried supernatant was weighed and recorded as M4.

The Water Solubility Index (WSI) and Swelling Power (SP) of the sample can be calculated using the following equations:

$$\mathrm{WSI},\%=\frac{M4-M3}{M}\times 00\%$$

$$\mathrm{SP},g/g=\frac{M2-M1}{M\times \left(1-\mathrm{WSI}\right)}$$

### 3.10. Molecular Weight Distribution and Amylopectin Chain-Length Analysis

Starch samples were deproteinized using a combination of protease and sodium bisulfite, then debranched with isoamylase following the method described by Lin et al. (2016) [43]. The molecular weight distribution of the debranched starch was analyzed using a U3000 high-temperature chromatograph (Thermo, Waltham, MA, USA) equipped with an Ohpak SB-805 HQ (300 × 8 mm) or Ohpak SB-803 HQ (300 × 8 mm) gel exclusion column appropriate to the molecular weight range of interest. The column temperature was maintained at 60 °C and a sample size of 100 μL was used. The mobile phase consisted of 0.5% LiBr in DMSO (mobile phase A) and the flow rate was set to 0.3 mL/min. The elution gradient was isocratic, with a total run time of 120 min.

The analysis of amylopectin branch chain length distribution of starches was conducted using a high-performance anion-exchange chromatography (HPAEC) system equipped with a pulsed amperometric detector (PAD) known as the Dionex ICS 5000 system. Assistance for gel permeation chromatography and the analysis of amylopectin chain length was provided by Sanshu Biotech Co., (Shanghai, China).

### 3.11. In Vitro Starch Digestion Characterization

The in vitro starch digestion of rice samples before and after roasting were determined based on the modified method of Bai et al. [39,44]. α-amylase was weighed (3 g) and added to a beaker along with 30 mL of distilled water. The mixture was then stirred using a magnetic stirrer for 5 min. After that, it was centrifuged at 1500× *g* for 10 min, and 18 mL of the supernatant was carefully transferred into another beaker.

Next, 2.0 mL of starch glucosidase was taken and diluted to 2.5 mL. From the diluted starch glucosidase, 2.0 mL was added to the beaker containing the α-amylase supernatant. Additionally, 25 mg of invertase was added to the beaker and thoroughly mixed. In parallel, 100 mg of crushed 80-mesh sieve material was weighed and added to a 50 mL centrifuge tube. This step was repeated three times. Subsequently, 2 mL of pepsin-guar gum solution was added, and the mixture was vigorously shaken using a vortex. To prevent agglomeration, 2 glass beads were added, and the tube was then placed in a water bath set to 37 °C with an oscillation speed of 150 rpm. The mixture was incubated for 30 min. After incubation, 4 mL of a 0.5 mol/L sodium acetate buffer solution was added to the centrifuge tube, and it was swirled and thoroughly mixed. The tube was incubated again in a water bath at 37 °C with an oscillation speed of 150 rpm for 5 min. Following this, 1 mL of a mixed enzyme solution was added. The contents of the tube were mixed using vortexing and placed in a water bath at 37 °C with an oscillation speed of 150 rpm. The timing was strictly observed, and samples were collected at intervals of 30, 60, 90, 120, 150 and 180 min. Specifically, 100 μL of the hydrolysate was obtained and transferred into a 2 mL centrifuge tube after vortex mixing. The obtained hydrolysate was then combined with 1 mL of a 50% ethanol solution (by volume fraction), mixed well, and centrifuged at 8000× *g* for 10 min. The released glucose amount was determined using a GODPOD 4058 assay kit (Giesse Diagnostic snc, Rome, Italy). The percentage of digested starch was calculated using a conversion factor of 0.9. The starch hydrolysis index (HI) was derived from the area under the starch hydrolysis curve (0–180 min) with white wheat bread [39]. Analyses were run in triplicate.

### 3.12. Statistical Analyses

Experimental data were analyzed using analysis of variance (ANOVA), expressed as the mean value ± SD, and significant differences among means were determined by Duncan’s test. All analyses were performed using the SPSS Statistics software ver.18 (SPSS Inc., Chicago, IL, USA). The significance level in all cases was set at *p* < 0.05.

## 4. Conclusions

We have successfully developed “Youtang Rice flour” by precisely controlling moisture levels and implementing traditional roasting techniques. This specialized rice flour product exhibits a resistant starch content exceeding 20%. Youtang Rice roasting flour maintains a lower starch digestion rate while simultaneously enhancing taste and texture. Consequently, it becomes an invaluable ingredient in the production of grain meal replacement powders. The findings contribute to improving the understanding of the properties and benefits of high-RS rice as a potential ingredient with enhanced nutritional value and functional properties in the food industry.

Although we have successfully developed the high-RS rice flour product. It is important to acknowledge its limitations and potential implications. These issues need to be addressed through further research and development in order to fully maximize the advantages of this product. Firstly, one limitation lies in controlling the moisture content. Research indicates that maintaining a moisture content between 10% and 20% can result in higher RS content, with 15% being considered the optimal level. However, ensuring stable and controlled moisture content in actual production may present challenges. Achieving consistent moisture levels may require the use of specialized techniques and equipment. Thus, further optimization of the production process is needed to ensure stable and controlled moisture content in each batch. Secondly, the roasting conditions play a crucial role in determining the final performance of the product. While we have utilized traditional roasting techniques to improve the texture and taste of Youtang Rice flour, variations in roasting conditions may yield different results. Further research can explore and optimize the roasting time, temperature, and other parameters to identify the optimal roasting conditions for achieving enhanced product quality. Additionally, despite highlighting its high RS content and heat stability, there may still be some RS losses during the cooking and puffing processes. Further investigations are required to maximize the retention of RS content and ensure that the developed rice flour can deliver the expected benefits in practical usage.

## Figures and Tables

**Table 1:** Moisture content, Resistant starch content, and Apparent amylose content of Different Samples after roasting.

Sample	MC (%)	RS (%)	AAC (%)
YTD2-100%	3.73 ± 0.15 a	12.78 ± 0.59 c	30.8 ± 0.44 a
YTD2-90%	3.86 ± 0.12 a	16.91 ± 0.36 b	31.5 ± 1.21 a
YTD2 -85%	3.75 ± 0.09 a	22.61 ± 1.21 a	31.2 ± 1.01 a
YTD2-80%	4.08 ± 0.13 a	17.75 ± 0.48 b	31.1 ± 0.56 a
YTD2-70%	4.15 ± 0.11 a	8.42 ± 0.15 d	28.1 ± 0.76 a
HDXR34-100%	3.85 ± 0.08 a	0.041 ± 0.01 e	11.1 ± 0.39 b
HDXR34-90%	3.95 ± 0.16 a	0.053 ± 0.03 e	11.2 ± 0.24 b
HDXR34-85%	4.32 ± 0.12 a	0.058 ± 0.05 e	11.5 ± 0.34 b
HDXR34-80%	3.88 ± 0.15 a	0.075 ± 0.06 e	11.4 ± 0.15 b
HDXR34-70%	4.29 ± 0.05 a	0.067 ± 0.01 e	11.6 ± 0.31 b

The analysis was repeated three times. Different lowercase letters represent the significant differences of values. *p* < 0.05 was considered as being significantly different. MC, Moisture content; RS, Resistant starch content; AAC, Apparent amylose content; YTD2, Youtangdao2; HDX34, Hudaoxiangruan34.

**Table 2:** Comparison of flavor substances of high-RS riceYTD2 before and after roasting.

Substance Category	Before Roasting		After Roasting	
	Quantity	Relative Content (%)	Quantity	Relative Content (%)
Acid	2	43.55	3	2.68
Alcohol	2	2.23	1	0.12
Ester	1	0.6	0	0
Aldehyde	1	13.56	3	17.29
Amino Acid	1	3.18	0	0
Amine	1	1.32	0	0
Olefin	2	2.49	0	0
Aromatic Hydrocarbon	1	0.46	1	0.46
Acid Anhydride	1	0.55	0	0
Fatty Acid	1	3.07	0	0
Pyrazine	0	0	8	29.43
Ketone	0	0	3	2.36
Aromatic Compound	0	0	3	3.54
Others	23	29.09	21	40.72

**Table 3:** DCS thermal stability test results of Rice Before and After Roasting.

Sample Name	ΔH (J/g)	Tp (°C)	To (°C)	Tc (°C)
Before roasting	2.9 ± 0.2	77.2 ± 0.5	67.0 ± 0.3	86.1 ± 0.5
After roasting	3.7 ± 0.3 **	85.4 ± 0.4 *	76.6 ± 0.4 *	92.8 ± 0.4 *

Tp represents the maximum peak temperature of particle thermal decomposition, To represents the initial temperature of particle thermal decomposition, Tc represents the high temperature thermal stability temperature of particles, ΔH represents the heat absorbed or released per gram of sample. * Significant difference, (0.01 < *p* < 0.05); ** Significant difference, (*p* < 0.01).

**Table 4:** The comparison of water solubility index and swelling power before and after roasting.

Sample Name	Water Solubility Index (%)	Swelling Power (g/g)
Before roasting	37.42.0 ± 0.3	13.55 ± 0.5
After roasting	24.63 ± 0.4 *	8.39 ± 0.4 **

* Significant difference, (0.01 < *p* < 0.05); ** Significant difference, (*p* < 0.01).

**Table 5:** The starch molecular weight distribution of high-RS rice before and after roasting.

Sample	Peak1 Area/Total Area	Peak2 Area/Total Area	Peak3 Area/Total Area
Before roasting	47.99% ± 0.3%	35.56% ± 0.2%	16.45% ± 0.1%
After roasting	52.51% ± 0.4%	31.49% ± 0.6%	16.0% ± 0.2%

## Data Availability

Data are contained within the article.

## Sample Availability

Samples of the high-RS rice varieties Youtangdao are available from the authors.
